# Diffusion-Based EM Algorithm for Distributed Estimation of Gaussian Mixtures in Wireless Sensor Networks

**DOI:** 10.3390/s110606297

**Published:** 2011-06-14

**Authors:** Yang Weng, Wendong Xiao, Lihua Xie

**Affiliations:** 1 School of Mathematics, Sichuan University, Chengdu 610064, China; E-Mail: wengyang@scu.edu.cn; 2 Institute for Infocomm Research, 138632, Singapore; 3 School of Electrical and Electronic Engineering, Nanyang Technological University, 639798, Singapore; E-Mail: elhxie@ntu.edu.sg

**Keywords:** diffusion, distributed processing, EM algorithm, consensus, wireless sensor networks

## Abstract

Distributed estimation of Gaussian mixtures has many applications in wireless sensor network (WSN), and its energy-efficient solution is still challenging. This paper presents a novel diffusion-based EM algorithm for this problem. A diffusion strategy is introduced for acquiring the global statistics in EM algorithm in which each sensor node only needs to communicate its local statistics to its neighboring nodes at each iteration. This improves the existing consensus-based distributed EM algorithm which may need much more communication overhead for consensus, especially in large scale networks. The robustness and scalability of the proposed approach can be achieved by distributed processing in the networks. In addition, we show that the proposed approach can be considered as a stochastic approximation method to find the maximum likelihood estimation for Gaussian mixtures. Simulation results show the efficiency of this approach.

## 1. Introduction

Recently, with the advances in micro-electronics and wireless communications, the large scale wireless sensor networks (WSNs) have found applications in many domains, such as environment monitoring, vehicle tracking, healthcare, *etc.* These WSNs usually consist of massively distributed low-cost, low-power and small size sensor nodes, which have sensing, data precessing and communication capabilities [1]. However due to limited sensor and network resources, energy-efficient collaborative signal processing algorithms are needed [2–4].

Mixture density is a powerful family of probability density to represent some physical quantities. The most wildly used mixture is Gaussian mixture model (GMM), which has wide applications in WSNs such as energy based multi-source localization and maneuvering target tracking [5,6]. Density estimation is the most important step in exploratory data analysis for such models. The well-known expectation-maximization (EM) algorithm has been extensively applied for such a purpose [7].

The EM algorithm is used for finding maximum likelihood estimates of parameters in probabilistic models, where the model depends on unobserved latent variables. The EM algorithm was explained and given its name in a classic 1977 paper by Arthur Dempster, Nan Laird, and Donald Rubin [8]. They pointed out that the method had been “proposed many times in special circumstances” by earlier authors. The EM algorithm is implemented by performing an expectation step (E-step), which computes an expectation of the likelihood by including the latent variables as if they were observed, and a maximization step (M-step), which computes the maximum likelihood estimates of the parameters by maximizing the expected likelihood found on the E-step. The parameters found on the M-step are then used to begin another E-step, and the process is repeated until converging to a local maximum for the likelihood.

The EM algorithm has been widely used in many areas, such as econometric, clinical, engineering and sociological studies [9,10]. However, most existing EM algorithms in WSNs are designed in a centralized way with a fusion center. Distributed algorithms have many advantages on robustness and scalability in large scale WSNs over the centralized scheme due to their distributed nature, node redundancy, and the lack of dangers when the fusion center fails. Robust, asynchronous and distributed algorithms have seen more and more opportunities as the intelligent and autonomous sensors play a more important role in the networks, as demonstrated in estimation, detection and control [11–13].

The distributed processing framework in WSNs depends on the strategies of cooperation that are allowed among the nodes. Two of the strategies used in the literature for distributed processing are the incremental strategy and the consensus strategy. In the incremental strategy, information flows in a sequential manner from one node to the other. This mode of operation requires a cyclic pattern of collaboration among the nodes [14]. This incremental idea is first introduced to distributed estimation in adaptive networks [15]. An incremental strategy-based EM algorithm for Gaussian mixtures in WSNs is proposed in [16]. In this algorithm, each node computes its local statistics. The algorithm obtains the global statistics along a pre-selected path by adding the local statistics of the node, then updates the estimate of each node in the reverse order of the path. Although it does not need a fusion center and tends to require the least amount of communications, usually there is a long way from the first node to the last node of the pre-selected path in a large scale WSNs that may cause serious reliability problem when any node along the path fails.

In the consensus strategy, distributed estimation algorithms are proposed in [17,18], where the consensus is implemented in two time scales: a slower time scale for obtaining measurements and a faster time scale for processing iterations. The fundamental objective in consensus-based algorithm is to obtain the same estimate for all nodes at each iteration. In [19], another consensus-based distributed EM algorithm for Gaussian mixtures is proposed. In this algorithm, a consensus filter (C-step), in which the global statistics for each sensor node is obtained by average consensus, is implemented between the E-step and the M-step at each iteration. However, in a large scale sensor network, the consensus for all the sensor nodes at each iteration can only be achieved with large amount of communications and consume a lot of battery power.

For the incremental strategy-based methods, finding the communication path through all nodes is the well-known Hamiltonian circuit problem, which is proved to be NP-complete [20]. The nature of two time scales in consensus-based methods limits their applications to WSNs which is resource constrained for communications [15]. Recently, a number of novel diffusion adaptation strategies are introduced for distributed estimation, detection and filtering in networked systems, including diffusion recursive least-squares estimation algorithm [21], diffusion least-mean squares estimation algorithms [22–24], diffusion adaption for distributed detection [25], and diffusion Kalman filtering and smoothing algorithm for dynamic system [26]. Different from the consensus strategy with two time scales among neighboring nodes, diffusion strategy only needs to update the estimate for each node by single time scale, which can therefore lead to good performance with less communication overhead.

In this paper, we propose a diffusion scheme of EM (DEM) algorithm for Gaussian mixtures in WSNs. At each iteration, the time-varying communication network is modeled as a random graph. A diffusion-step (D-step) is implemented between the E-step and the M-step. In the E-step, sensor nodes compute the local statistics by using local observation data and parameters estimated at the last iteration. In the D-step, each node exchanges local information only with its current neighbors and updates the local statistics with exchanged information. In the M-step, the sensor nodes compute the estimation of parameter using the updated local statistics by the D-step at this iteration. Compared with the existing distributed EM algorithms, the proposed approach can extensively save communication for each sensor node while maintain the estimation performance. Different from the linear estimation methods such as the least-squares and the least-mean squares estimation algorithms, each iteration of EM algorithm is a nonlinear transform of measurements. The steady-state performance of the proposed DEM algorithm can not be analyzed by linear way. Instead, we show that the DEM algorithm can be considered as a stochastic approximation method to find the maximum likelihood estimation for Gaussian Mixtures.

The rest of this paper is organized as follows. In Section 2, the centralized EM algorithm for Gaussian mixtures in WSNs is introduced, and two distributed strategies for EM algorithms are reviewed. In Section 3, a randomly time-varying network is introduced and the DEM algorithm in WSNs is proposed. Section 4 discusses the asymptotical property of the proposed DEM algorithm, and shows that the proposed algorithm can be viewed as a stochastic approximation to the maximum likelihood estimator according to the *Robbins-Monro stochastic approximation* theory. Simulation results are presented in Section 5 to show the performance of our method. Concluding remarks are made in Section 6.

## 2. EM Algorithm for Gaussian Mixtures

We consider a network of *M* sensor nodes, each of which has *N_m_* data observations {*y_mn_*}, *m* = 1, 2, ···, *M*, *n* = 1, 2, ···, *N_m_*. These observations are drawn from a *K* Gaussian mixtures with mixture probabilities *α*_1_, ···, *α_K_*,

(1) $$y_{mn}∼\sum_{j=1}^{K}\alpha_{j}\cdot N(\mu_{j},\Sigma_{j})$$

where *N*(*μ*, ∑) denote the Gaussian density function with mean *μ* and covariance ∑. Let *z* ∈ {1, 2, ···, *K*} denote the missing data where Gaussian *y* comes from. The probability that a particular observation comes from the i-th dimensional Gaussian is

$$P\{y|z=i,\theta \}={\left(2\pi \right)}^{-D/2}{\left|\Sigma_{i}\right|}^{-1/2}\text{exp}\{-\frac{1}{2}{\left(y-\mu_{i}\right)}^{T}\Sigma_{i}^{-1}(y-\mu_{i})\}$$

Our task is using the observations to estimate the unknown parameters

$$\theta =\{\mu_{1},\cdots ,\mu_{K},\Sigma_{1},\cdots ,\Sigma_{K},\alpha_{1},\cdots ,\alpha_{K}\}$$

that is, the mean and standard deviation of each Gaussian and the probability for each Gaussian being drawn for any given point. For the centralized scheme for the EM algorithm, observations are transmitted to the central fusion center for executing the standard EM with all data observations.

The EM algorithm seeks to find the maximum likelihood estimation of the marginal likelihood by iteratively applying the E-step and M-step. In the E-step, we calculate the expectation of the log likelihood function, with respect to the conditional distribution of the unobserved data *z* given observations under the current estimate of the parameters *θ^t^*,

$$p(z_{mn}=i|y_{mn},\theta^{t})=\frac{p(z_{mn}=i,y_{mn}|\theta^{t})}{p(y_{mn}|\theta^{t})}=\frac{p(y_{mn}|z_{mn}=i,\theta^{t})p(z_{mn}=i|\theta^{t})}{\sum_{k=1}^{K}p(y_{mn}|z_{mn}=k,\theta^{t})p(z_{mn}=k|\theta^{t})}$$

where *θ^t^* means the estimation of parameters at the *t*-th iteration of the EM algorithm. Define the expected log-likelihood of the joint event:

$$\begin{array}{lll} Q(\theta ,\theta^{t}) & = & E_{z}\{\text{ln}[\prod_{m=1}^{M}\prod_{n=1}^{N_{m}}p(y_{mn},z|\theta )]|y_{mn},\theta^{t}\}\\ & = & E_{z}\{\sum_{m=1}^{M}\sum_{n=1}^{N_{m}}\;\text{ln}p(y_{mn},z|\theta )|y_{mn},\theta^{t}\}\\ & = & \sum_{m=1}^{M}\sum_{n=1}^{N_{m}}E_{z}\{\text{ln}p(y_{mn},z|\theta )|y_{mn},\theta^{t}\}\\ & = & \sum_{m=1}^{M}\sum_{n=1}^{N_{m}}p(z_{mn}=i|y_{mn},\theta^{t})\;\text{ln}p(z_{mn}=i,y_{mn}|\theta )\\ & = & \sum_{m=1}^{M}\sum_{n=1}^{N_{m}}p(z_{mn}=i|y_{mn},\theta^{t})\;\text{ln}[p(y_{mn}|z_{mn}=i,\theta )p(z_{mn}=i|\theta )] \end{array}$$

In the M-step, we want to maximize the expected log-likelihood *Q*(*θ*, *θ^t^*) and obtain the estimation of next step for *θ*, *i.e.*, *θ*^*t*+1^,

$$\theta^{t+1}=\text{arg}\;\underset{\theta }{\text{max}}Q(\theta ,\theta^{t})$$

By solving this maximization problem, we can get the estimation of *i*-th component’s parameters for the Gaussian mixtures at step *t* + 1:

(2) $$\begin{array}{lll} \mu_{i}^{t+1} & = & \frac{\sum_{m=1}^{M}\sum_{n=1}^{N_{m}}p(z_{mn}=i|y_{mn},\theta^{t})y_{mn}}{\sum_{m=1}^{M}\sum_{n=1}^{N_{m}}p(z_{mn}=i|y_{mn},\theta^{t})}\\ \sum_{i}^{t+1} & = & \frac{\sum_{m=1}^{M}\sum_{n=1}^{N_{m}}p(z_{mn}=i|y_{mn},\theta^{t})\;(y_{mn}-\mu_{i}^{t+1})\;{\left(y_{mn}-\mu_{i}^{t+1}\right)}^{T}}{\sum_{m=1}^{M}\sum_{n=1}^{N_{m}}p(z_{mn}=i|y_{mn},\theta^{t})}\\ \alpha_{i}^{t+1} & = & \frac{1}{N_{1}+\cdots +N_{M}}\sum_{m=1}^{M}\sum_{n=1}^{N_{m}}p(z_{mn}=i|y_{mn},\theta^{t}) \end{array}$$

The centralized EM algorithm enables the calculation of the global solution using all the observation data from each sensor in the network. We can rewrite Equation (2) into “sensor form” by introducing *membership function* and *local statistics* as follows,

- *Membership Function*
  (3) $$\varepsilon_{mn,i}^{t}=p(z_{mn}=i|y_{mn},\theta^{t-1})$$
  The membership function is the probability of which the observation data *y_mn_* belongs to *i*-th component of the Gaussian Mixtures.
- *Local Statistics*
  (4) $$\varepsilon_{m,i}^{t}=\sum_{n=1}^{N_{m}}\varepsilon_{mn,i}^{t},\beta_{m,i}^{t}=\sum_{n=1}^{N_{m}}\varepsilon_{mn,i}^{t}y_{mn,}\gamma_{m,i}^{t}=\sum_{n=1}^{N_{m}}\varepsilon_{mn,i}^{t}(y_{mn}-\mu_{i}^{t})\;{\left(y_{mn}-\mu_{i}^{t}\right)}^{T}$$

The estimation of parameters at the *t* + 1 step in Equation (2) can be rewritten as follows:

(5) $$\mu_{i}^{t+1}=\frac{\sum_{m=1}^{M}\beta_{m,i}^{t+1}}{\sum_{m=1}^{M}\varepsilon_{m,i}^{t+1}},\sum_{i}^{t+1}=\frac{\sum_{m=1}^{M}\gamma_{m,i}^{t+1}}{\sum_{m=1}^{M}\varepsilon_{m,i}^{t+1}},\alpha_{i}^{t+1}=\frac{1}{N_{1}+\cdots +N_{M}}\sum_{m=1}^{M}\varepsilon_{m,i}^{t+1}$$

We denote ${ɛ}_{i}^{t+1}=\sum_{m=1}^{M}{ɛ}_{m,i}^{t+1},\beta_{i}^{t+1}=\sum_{m=1}^{M}\beta_{m,i}^{t+1},\gamma_{i}^{t+1}=\sum_{m=1}^{M}\gamma_{m,i}^{t+1}$ as the *global statistics*. Since the *Local Statistics* $\left\{{ɛ}_{m,i}^{t},\beta_{m,i}^{t},\gamma_{m,i}^{t}\right\}$ can be calculated locally given the current estimated parameter set *θ^t^* and the local observation data *y_mn_*, there are two distributed strategies for implementation of the EM algorithm in WSNs.

1. Incremental Updating Strategy
  In this strategy, a cyclic sequential communication path between sensor nodes is pre-determined. In the forward path, the global statistics is updated by the pre-selected path for all sensor nodes according to
  $${ɛ}_{i}^{t}={ɛ}_{i}^{t}+{ɛ}_{m,i}^{t},\beta_{i}^{t}=\beta_{i}^{t}+\beta_{m,i}^{t},\gamma_{i}^{t}=\gamma_{i}^{t}+\gamma_{m,i}^{t}$$
  In the backward path, each node updates the parameter set *θ^t^*, which is the global solution at the M-step, according to Equation (5) in the reverse order.
2. Consensus Strategy
  The estimation of parameter set *θ^t^* in Equation (5) is not changed at the M-step. If we redefine the global statistics $\left\{{ɛ}_{i}^{t},\beta_{i}^{t},\gamma_{i}^{t}\right\}$ as the averages of the local statistics $\left\{{ɛ}_{m,i}^{t},\beta_{m,i}^{t},\gamma_{m,i}^{t}\right\}$, then
  (6) $$\mu_{i}^{t+1}=\frac{\frac{1}{M}\sum_{m=1}^{M}\beta_{m,i}^{t+1}}{\frac{1}{M}\sum_{m=1}^{M}\varepsilon_{m,i}^{t+1}},\sum_{i}^{t+1}=\frac{\frac{1}{M}\sum_{m=1}^{M}\gamma_{m,i}^{t+1}}{\frac{1}{M}\sum_{m=1}^{M}\varepsilon_{m,i}^{t+1}},\alpha_{i}^{t+1}=\frac{M}{N_{1}+\cdots +N_{M}}\frac{1}{M}\sum_{m=1}^{M}\varepsilon_{m,i}^{t+1}$$
  Therefore, the idea of the average consensus filter can be used after the computing local statistics at the E-step. Each sensor node can get the global statistics by exchanging the local statistics with its neighbors until reaching consensus all over the network. The global solution for each sensor node at the M-step can be obtained by Equation (6).

For both strategies, individual sensor node first computes its own local statistics using the local observation data and estimation of the last step, and then gets the global statistics or global estimation for the parameters by changing information with its neighbors in some specific way. Therefore, each node can obtain the estimation of the parameters equivalent to the estimation in centralized way at every step, and get the same convergence point as the standard EM.

## 3. Diffusion Scheme of EM Algorithm for Gaussian Mixtures in WSNs

Both of the strategies described in Section 2 can be adopted to implement the EM algorithm in distributed way. However, as noted earlier in [15], there are several disadvantages for both methods. For the *incremental strategy*, the communication path should be determined before the implementation. Each sensor node should assign a subsequent node to form a circle containing all the sensor nodes in the network at first. The communication will then start from one node to its subsequent node and relay the information sequentially. At the last update, the global estimation for the parameter is generated in the reverse order. For a large scale sensor network, this strategy is untractable since the last node will wait a long time to receive the information from its previous node and then the global estimation for the parameter will need a long way to transmit to every node as well. This approach will fail when any node in the network is shut down or run out of energy.

For the *Consensus strategy*, a consensus filter will be implemented after the E-step at each iteration, namely C-step (consensus step). In this step, each sensor node will exchange local information with its neighbors until the agreement is reached. R. Carli *et al.* pointed out that the time-invariant communication networks with symmetries yield slow convergence to consensus, as the convergence rate will degrade as the number of sensor nodes increase, if the amount of information exchanged by the nodes does not scale well with their total number [27]. In a large scale WSN, the consensus of global statistics for all the sensor nodes requires quite a bit of communications to the neighbors and consume a lot of battery power.

Consider a WSN in which each sensor node has local computation functions and can only communicate with sensors in its neighborhood. The network can be modeled as a graph *𝒢*(*V*, *E*) with the vertex set *V* = {1, ···, *n*} and the edge set *E* = {(*i*, *j*) ∈*V* × *V* }. Each sensor node is a vertex of the graph. There is an edge between sensor nodes *i* and *j* if their distance is less than or equal to the radio range *r*, *i.e.*, *E* = {(*i*, *j*)|*d_ij_* ≤ *r*}. We assume the graph is connected, *i.e.*, there exists a path in *E* for any two vertices. The set of neighbors of node *i* is defined as the nodes which can communicate to node *i* directly, *i.e.*, *𝒩_i_* = {*j*|(*i*, *j*) ∈ *E*}.

Power consumption of the sensor nodes in WSN can be divided into three domains: sensing, communication, and data processing [1]. Among them, usually a sensor node spends much more energy in data communication than sensing and data processing. In this paper, we also make such assumption. Therefore, an important aspect for performing tasks in a distributed fashion in WSN is to cap the communication cost. The communication cost of the topology *𝒢*(*V*, *E*) can be defined as a function of the adjacency elements by

$$𝒞=\sum_{i,j=1}^{n}sgn(a_{ij})$$

where *sgn*(·) is the sign function and *a_ij_* is the nonnegative adjacency element of adjacency matrix *A* of topology *𝒢*(*V*, *E*) [13]. An adjacency matrix is a means of representing which vertices of a graph are adjacent to which other vertices. Specifically, the adjacency matrix of a finite graph *𝒢*(*V*, *E*) on *n* vertices is the *n* × *n* matrix where the non-diagonal entry *a_ij_* is the number of edges from vertex *i* to vertex *j*, According to this definition, *𝒞* is the same as |*E*| for a digraph.

By the definition of communication cost, the incremental strategy tends to require the least amount of communications. However, it inherently requires a Hamiltonian cycle through which signal estimates are sequentially circulated from sensor to sensor. In addition, the eventuality of a sensor failure also challenge the applicability of incremental schemes in large scale WSNs. The major disadvantage of the consensus-based algorithm is its high communication overhead requirements of each node. Motivated by these results and by diffusion strategies of [21–26], we propose a diffusion distributed EM algorithm with *D-step* (diffusion-step) between E-step and modified M-step. In such algorithm each sensor node exchanges local information with its neighboring nodes only once after the local statistics has been computed based on the local data at each iteration in EM algorithm. In the E-step, sensor nodes compute the local statistics by using local observation data and parameters estimated at the last iteration. In the D-step, each node exchanges local information only with its current neighbors. In the M-step, the sensor nodes communicate their local preestimates with their neighbors and perform average operations to obtain the estimation of parameters at this iteration. This DEM algorithm is summarized as follows,

1. E-step
  At time t, the nodes compute their local statistics with local observation data and the estimated parameter at the last step as follows,
  (7) $$\varepsilon_{m,i}^{t}=\sum_{n=1}^{N_{m}}\varepsilon_{mn,i}^{t},\beta_{m,i}^{t}=\sum_{n=1}^{N_{m}}\varepsilon_{mn,i}^{t}y_{mn},\gamma_{m,i}^{t}=\sum_{n=1}^{N_{m}}\varepsilon_{mn,i}^{t}(y_{mn}-\mu_{i}^{t})\;{\left(y_{mn}-\mu_{i}^{t}\right)}^{T}$$
2. D-step
  Each node communicates its local statistics with its neighbors as follows,
  (8) $$\varepsilon_{m,i}^{t}=\frac{1}{\left|{𝒩}_{i}\right|}\sum_{l\in {𝒩}_{m}}\varepsilon_{l,i}^{t},\beta_{m,i}^{t}=\frac{1}{\left|{𝒩}_{i}\right|}\sum_{l\in {𝒩}_{m}}\beta_{l,i}^{t},\gamma_{m,i}^{t}=\frac{1}{\left|{𝒩}_{i}\right|}\sum_{l\in {𝒩}_{M}}\gamma_{l,i}^{t}$$
3. M-step
  Each node computes the pre-estimation and communicates it to its neighbors to perform the average according to
  (9) $$\mu_{m,i}^{t}=\frac{1}{\left|{𝒩}_{i}\right|}\sum_{l\in {𝒩}_{m}}\mu_{l,i}^{t},\Sigma_{m,i}^{t}=\frac{1}{\left|{𝒩}_{i}\right|}\sum_{l\in {𝒩}_{m}}\Sigma_{l,i}^{t},\alpha_{m,i}^{t}=\frac{1}{\left|{𝒩}_{i}\right|}\sum_{l\in {𝒩}_{m}}\alpha_{l,i}^{t}$$
  where *𝒩_i_* denote the current neighbor set of node *m*.

In our proposed method, each sensor node needs to transmit the local statistics and preestimates to neighboring nodes at each iteration while the distributed EM with consensus strategy need much more amount of communication to achieve consensus, especially in large scale WSNs. Without loss of generality, we consider the one-dimensional Gaussian mixtures with *K* components. Each node has 3*K* scalars of local statistics ( $\left\{{ɛ}_{m,i}^{t},\beta_{m,i}^{t},\gamma_{m,i}^{t}\right\}$, *i* = 1 ··· *K*) that need to be transmitted. In the incremental strategy, the communication complexity per node is 3*K* = *𝒪*(*K*), since the local statistics only need to be transmitted to the next node. In the consensus strategy, the communication complexity per node is *𝒪*(|*𝒩_i_*| · *K*^2^), since the local statistics need to be exchanged between all of the neighbors until they achieve consensus [15,17]. The communication complexity per node for the diffusion strategy is |*𝒩_i_*| · 3*K* = *𝒪*(|*𝒩_i_*| · *K*), which is much lower than the consensus strategy.

## 4. Performance Analysis

An important question is how well does the proposed DEM algorithm perform the estimation task. That is, how close does the local estimate get to the desired parameter as time evolves. Analyzing the performance of such diffusion scheme is challenging since each node is influenced by the local statistics from the neighboring nodes. In this section, we analyze the performance of the proposed DEM algorithm and show that it can be considered as a stochastic approximation method to find the maximum likelihood estimator. That is, if an infinite number of data, which are drawn independently according to the probability distribution *ρ*(*y*), are available for each sensor, the DEM algorithm can be considered as a stochastic approximation for obtaining the maximum likelihood estimation of the Gaussian mixtures for all the sensor nodes in the networks. Under some rough conditions, the maximum likelihood estimator is consistent, which means that as the number of data goes to infinity the estimator converges in probability to its true value. The main result of this section will be concluded in Theorem 1 at the end of this section.

First, we introduce a symbol *〈*·*〉_i_* below to denotes a weighted mean with respect to the posterior probability that proposed in Equation (3) as a membership function for each observation data,

(10) $${〈f(y)〉}_{i}(N)=\frac{1}{N}\sum_{n=1}^{N}f(y_{n})p(i|y_{n},\theta )$$

where *i* denotes the i-th component of Gaussian Mixtures and N denotes the number of all data. Under this notation, the M-step in Equation (2) can be written as

(11) $$\alpha_{i}={〈1〉}_{i}(N),\mu_{i}=\frac{{〈y〉}_{i}(N)}{{〈1〉}_{i}(N)},\Sigma_{i}=\frac{{〈(y-\mu_{i})\;(y-\mu_{i})\prime 〉}_{i}(N)}{{〈1〉}_{i}(N)}$$

If an infinite number of data are drawn independently according to the unknown probability distribution density *ρ*(*y*), the weighted mean converges to the following expectation value,

(12) $${〈f(y)〉}_{i}(N)\to E{\left[f(y_{t})p(i|y_{t},\theta )\right]}_{\rho },N\to \infty$$

where *E*[·]*_ρ_* denotes the expectation with respect to the probability distribution density *ρ*(*y*). In this case, the M-step at the t-th iteration can be written as

$$\alpha_{i}^{t+1}=E{\left[p(i|y,\theta^{t})\right]}_{\rho },\mu_{i}^{t+1}=\frac{E{\left[yp(i|y,\theta^{t})\right]}_{\rho }}{E{\left[p(i|y,\theta^{t})\right]}_{\rho }},\Sigma_{i}^{t+1}=\frac{E{\left[(y-\mu_{i}){\left(y-\mu_{i}\right)}^{\prime }p(i|y,\theta^{t})\right]}_{\rho }}{E{\left[p(i|y,\theta^{t})\right]}_{\rho }}$$

At an equilibrium point of this EM algorithm, the estimated parameter at the last step and current step becomes the same: *θ^t^* = *θ*^*t*+1^. The equilibrium condition of this EM algorithm is equivalent to the maximum likelihood condition,

(13) ∂L(θ)/∂θ=0

where the log-likelihood function *L*(*θ*) is defined as:

(14) $$L(\theta )=E{\left[\text{log}\left(\sum_{i=1}^{K}\alpha_{i}p(y|\theta )\right)\right]}_{\rho }$$

Since we consider the asymptotical property of the DEM algorithm, one more data can be taken at each iteration. Therefore, the local standard EM algorithm for each sensor node is a modified on-line version of the EM algorithm [28]. In the on-line EM algorithm, the weighted mean defined in Equation (10) is replaced by

(15) $$\begin{array}{lll} {〈〈f(y)〉〉}_{i}(N) & = & \eta (N)\sum_{n=1}^{N}\left(\prod_{m=n+1}^{N}\lambda (s)\right)\times f(y_{n})p(i|y_{n},\theta n-1)\\ \eta (N) & = & ={\left(\sum_{n=1}^{N}\prod_{m=n+1}^{N}\lambda (s)\right)}^{-1} \end{array}$$

where the parameter *λ*(*m*), 0 ≤ *λ*(*s*) ≤ 1 is a time dependent forgetting factor, which is introduced for forgetting the effect of the former observation to the current estimator. The parameter *η*(*n*) is a normalization coefficient.

The modified weighted mean *〈〈*·*〉〉_i_*, which is proposed in Equation (15), can be written as the step-wise version as follows:

(16) $$\begin{array}{lll} {〈〈f(y)〉〉}_{i}(t) & = & {〈〈f(y)〉〉}_{i}(t-1)+\eta (t)\{f(y_{t})p_{i}(t)-{〈〈f(y)〉〉}_{i}(t-1)\}\\ \eta (t) & = & {\left(1+\lambda (t)/\eta (t-1)\right)}^{-1} \end{array}$$

where *p_i_*(*t*) = *p*(*i|y_t_*, *θ*^*t*−1^), and *θ^t^* denotes the estimation of parameter after *t* iterations with *t* observations.

After computing the modified weighted means according to Equation (16), the current estimation of parameter *θ^n^* can be written as:

(17) $$\alpha_{i}^{t}={〈〈1〉〉}_{i}(t),\mu_{i}^{t}=\frac{{〈〈y〉〉}_{i}(t)}{{〈〈1〉〉}_{i}(t)},\Sigma_{i}^{t}=\frac{{〈〈(y-\mu_{i})\;{\left(y-\mu_{i}\right)}^{\prime }〉〉}_{i}(t)}{{〈〈1〉〉}_{i}(t)}$$

In the basic on-line EM algorithm, for the t-th observation *y_t_*, the weighted means are calculated by using the step-wise Equation (16), and the current estimation of parameter is computed according to Equation (17). It can be shown that the on-line EM algorithm is equivalent to the standard batch EM algorithm if a specific assignment of the forgetting factor is employed [28].

For the WSN case, the weighted means are actually the local statistics, which are denoted as

$$\varphi_{m,i}^{t}=\{{〈〈1〉〉}_{i}(t),{〈〈y〉〉}_{i}(t),{〈〈(y-\mu_{i})\;{\left(y-\mu_{i}\right)}^{\prime }〉〉}_{i}(t)\}$$

where *m* denotes the m-th sensor node and *i* denotes the i-th component of the Gaussian Mixtures. The local on-line EM algorithm for the m-th sensor node can be written in an abstract form according to Equations (16) and (17):

(18) $$\begin{array}{lll} \delta \varphi_{m}^{t} & = & \varphi_{m}^{t}-\varphi_{m}^{t-1}=\eta (t)[F(y,\theta^{t-1})-\varphi_{m}^{t-1}]\\ \;\theta_{m}^{t} & = & H(\varphi_{m}^{t}) \end{array}$$

where $\theta_{m}^{t}$ denotes the estimation of the parameters for m-th node at time t, and $\varphi_{m}^{t}=\{\varphi_{m,1}^{t},\cdots ,\varphi_{m,K}^{t}\}$ denotes the local statistics for all components.

A distributed dynamic system consists of *M* dynamic elements over an information networks can be described in the following:

$${\dot{z}}_{m}^{t}=\sum_{l\in {𝒩}_{m}}(z_{l}^{t}-z_{m}^{t})\;m=1,\cdots ,M$$

where *𝒩_m_* denotes the set of neighboring nodes for node *m* in the network. This system can be written as a matrix form

(19) $${\dot{z}}^{t}=-Lz^{t}$$

where the L is the graph Laplacian, and we refer to Equation (19) as a first-order *Laplacian dynamics*.

The D-step of DEM algorithm, which is proposed in the previous section, can be formulated by the following Laplacian dynamics in the discrete form

(20) $$\varphi_{m}^{t}=\varphi_{m}^{t-1}+\eta (t)\left(\sum_{l\in {𝒩}_{m}}\left(\varphi_{l}^{t-1}-\varphi_{m}^{t-1}\right)\right)$$

Therefore, the abstract form of standard on-line EM algorithm proposed in Equation (18) can be replaced by

(21) $$\begin{array}{lll} \delta \varphi_{m}^{t} & = & \varphi_{m}^{t}-\varphi_{m}^{t-1}=\eta (t)[F(y,\theta^{t-1})-\varphi_{m}^{t-1}+\sum_{n\in {𝒩}_{m}}\left(\varphi_{n}^{t-1}-\varphi_{m}^{t-1})\right]\\ \theta_{m}^{t} & = & H(\varphi_{m}^{t}) \end{array}$$

From Equation (12), it can be easily proved that the following equations hold

(22) $$\begin{array}{l} \varphi_{m}=E{\left[F(y,\theta )\right]}_{\rho }\\ \theta_{m}=H(\varphi_{m}) \end{array}$$

which are equivalent to the maximum likelihood condition in Equation (13). Thus, the diffusion on-line EM algorithm can be written as,

(23) $$\delta \varphi_{m}^{t}=\eta (t)\;(E{\left[F(y,H(\varphi_{m}^{t-1}))\right]}_{\rho }-\varphi_{m}^{t-1}+\eta (t)\zeta (y_{t},{\bar{\varphi }}^{t-1})$$

where ${\bar{\varphi }}^{t-1}=\{\varphi_{l}^{t-1},l\in {𝒩}_{m}^{t-1}\}$, and the stochastic noise term *ζ* is defined by

(24) $$\zeta (y_{t},{\bar{\varphi }}^{t-1})=F(y_{t},H(\varphi_{m}^{t-1}))-E{\left[F(y,H(\varphi_{m}^{t-1}))\right]}_{\rho }+\sum_{l\in {𝒩}_{m}}\left(\varphi_{l}^{t-1}-\varphi_{m}^{t-1}\right)$$

Equation (23) has the same form as the *Robbins-Monro stochastic approximation* [29], which finds the maximum likelihood estimation given by Equation (22), as well as Equation (13). Therefore, the estimation of the parameter for each sensor node reaches the same maximum likelihood point asymptotically with the diffusion strategy for local statistics embedded in the standard EM algorithm.

For the convergence proof of the stochastic approximation in Equation (23), several conditions about the *stochastic noise* and the *coefficients* should be considered. It is easy to verify that the expectation of the stochastic noise satisfies,

(25) $$E[\zeta (y,\varphi_{m})]=0$$

and the noise variance *Var*[*ζ*]*_ρ_* is given by,

(26) $$Var{\left[\zeta (y_{t},{\bar{\varphi }}^{t-1})\right]}_{\rho }\leq 2Var{\left[F(y,H(\varphi_{m}))\right]}_{\rho }+2Var{\left[\sum_{l\in {𝒩}_{m}}\left(\varphi_{l}^{t-1}-\varphi_{m}^{t-1}\right)\right]}_{\rho }$$

Both terms on the right side of Equation (26) are bounded if we assume the data distribution density *ρ*(*y*) has a compact support.

The normalized coefficient *η*(*t*) should satisfy the conditions,

(27) $$\eta (t)\to 0,t\to \infty ,\sum_{t=1}^{\infty }\eta (t)=\infty ,\sum_{t=1}^{\infty }\eta^{2}(t)<\infty$$

Recalling the definition of the coefficients *λ*(*t*) and *η*(*t*) in Equations (15) and (16), we have the constraints,

(28) 0≤λ(t)≤1,1/t≤η(t)≤1

Therefore, we define the *η*(*t*) as,

(29) $$\eta (t)=\frac{1}{at},0<a<1$$

which satisfies the constraints in Equations (27) and (28).

**Theorem 1** *If an infinite number of data, which are drawn independently according to the Gaussian mixture model (1), are available, the proposed DEM algorithm (7), (8), (9) can be considered as a stochastic approximation for obtaining the maximum likelihood estimator of the desired parameters.*

## 5. Simulations

**Figure 1.**
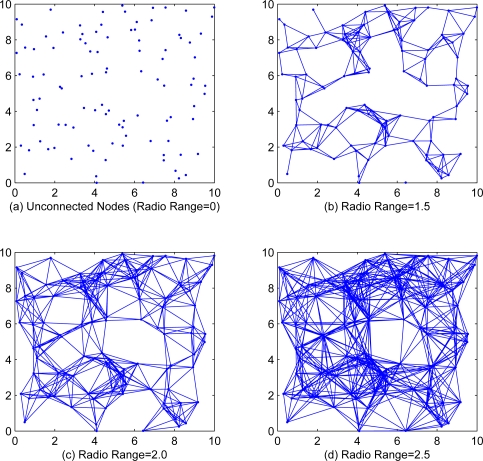
The connected network of 100 randomly distributed sensors with different radio ranges.

In this section, we will present simulation results to illustrate the effectiveness of our proposed algorithm. We randomly generate *n* = 100 sensor nodes in a 10 × 10 square, each sensor node takes 100 observation data. Two nodes will be connected with an edge while the distance between them is less than the communication range *r* of each node. Figure 1 shows the cases of different communication range with *r* = 0, *r* = 1.5, *r* = 2.0 and *r* = 2.5. Since the incremental strategy EM algorithm is intractable for medium and large scale WSNs, we only investigate the local, diffusion and consensus EM algorithms in simulations.

### 5.1. DEM Algorithm with 1-Dimensional Data

First, we consider the 1-dimensional Gaussian mixtures with two components. Each of the Gaussian components is with mean 6 and 15, variance 1 and 9, respectively. In the first 50 nodes (node 1 to node 50), 80% observations come from the first Gaussian component, and the rest 20% are from the second component. In the last 50 (node 51 to node 100), 40% observations come from the first component while the rest 60% are from the second one. The communication range is set to be 1.5 in this simulation.

**Figure 2.**
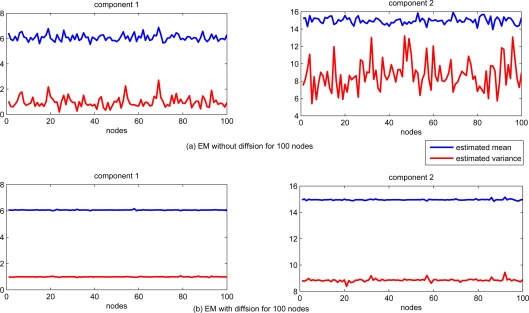
Estimated mean and covariance with and without diffusion.

The performance of estimation for each sensor node is shown in Figure 2. For comparison, the EM algorithm with and without information exchanging (local standard EM) are tested. The estimates of both mean and variance are significantly different for each sensor node with standard EM algorithm only based on the local data, while the estimation of both mean and variance with diffusion distributed EM algorithm are almost the same for each sensor node.

**Figure 3.**
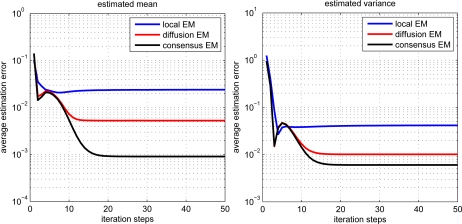
Performance comparison for different EM algorithms.

**Figure 4.**
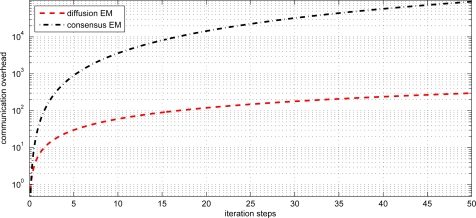
Communication overhead for diffusion EM and consensus EM.

**Figure 5.**
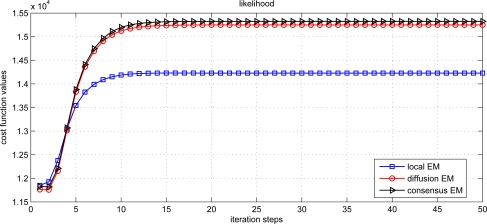
Likelihood function for different EM algorithms.

The estimation performance for local, diffusion and consensus EM algorithms is demonstrated in Figure 3. The average estimation error (AVE) is defined as

(30) $$\text{AVE}=\frac{1}{n}\sum_{i=1}^{n}\left\|{\hat{\theta }}_{i}-\theta \right\|$$

where *n* denotes the number of sensor nodes, *θ* denotes the desired parameter and *θ̂_i_* denotes the parameter estimate for the i-th node. From the figure, we can find that the local EM has the worst estimation performance. The diffusion EM is slightly worse than the consensus EM algorithm but with much less communication overhead, as depicted in Figure 4. This will lead to conserve energy and prolong the system lifetime. The values of log-likelihood function

(31) $$Q\;(\theta )=E_{z}\{\text{ln}[\prod_{m=1}^{M}\prod_{n=1}^{N_{M}}p(y_{mn},z|\theta )]|y_{mn}\}$$

are also shown in Figure 5. The likelihood for diffusion EM converges to almost the same value as the consensus EM.

**Figure 6.**
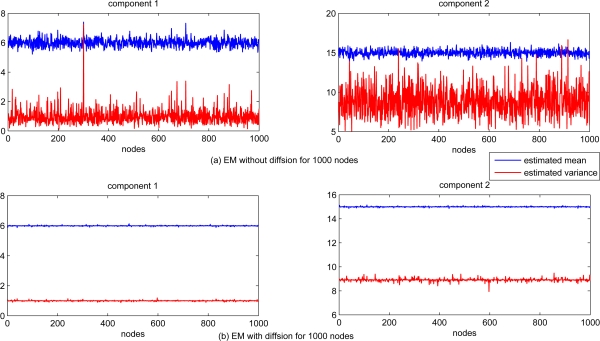
Estimated mean and covariance with and without diffusion.

**Figure 7.**
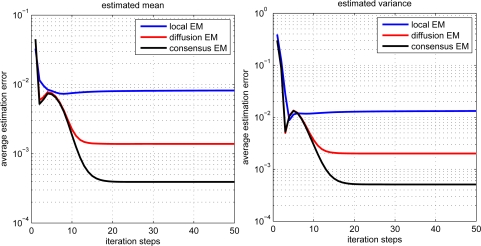
Performance comparison for different EM algorithms with 1,000 sensor nodes.

The scalability of the proposed diffusion EM algorithm is also investigated, with *n* = 1, 000 sensor nodes randomly generated in the same square. Each sensor node still takes 100 observation data and the radio range is set to 0.2. The estimated mean and variance values for all nodes are depicted in Figure 6 for both local EM and diffusion EM. The estimation performance for local, diffusion and consensus EM are demonstrated in Figure 7. we can still find that the local EM has the worst estimation performance while diffusion EM is slightly worse than the consensus EM algorithm.

### 5.2. DEM Algorithm with 2-Dimensional Data

**Figure 8.**
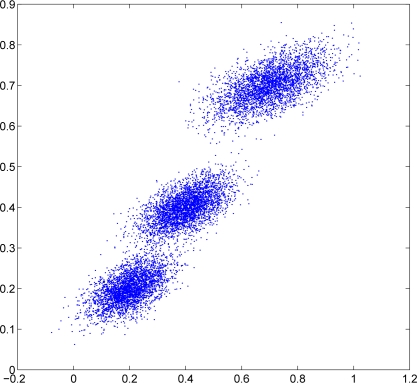
Data distribution with 3-components Gaussian mixtures.

In this subsection, we consider the 2D Gaussian density with *n* = 100 sensor nodes randomly generated in the same square. Each sensor node still takes 100 observation data. The observations are generated from 2D Gaussian mixtures with three components distributed in Figure 8. The observations for each sensor node are collected as follows. In the first 30 nodes (node 1 to node 30), 90% observations come from the first Gaussian component while the rest 10% observations come evenly from the other two Gaussian components. In the next 40 nodes (node 31 to node 70), 80% observations come from the second Gaussian component while the rest 20% observations come evenly from the other two Gaussian components. In the last 30 nodes (node 71 to node 100), 90% observations come from the third Gaussian component while the rest 10% observations come evenly from the other two Gaussian components.

**Figure 9.**
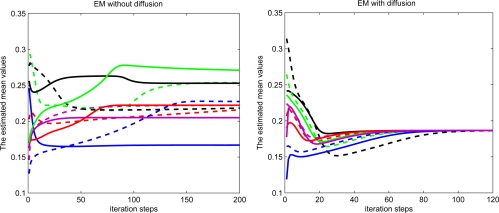
Estimated mean values by EM with and without diffusion.

The first estimated mean values during the iteration process for ten random selected sensor nodes are demonstrated in Figure 9. The radio range is set to 2 for each sensor. The estimated mean values almost converge to the same value for diffusion EM while the estimated mean values converge to different values for local EM algorithm.

**Figure 10.**
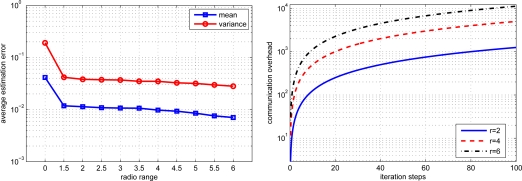
Estimation performance and communication overhead versus radio range.

We also investigated the impact of estimation performance and communication overhead with different communication ranges. Since local processing/computing is much less expensive than communication, we emphasize on the tradeoff between estimation performance and communication overhead. From Figure 10 we can see that increasing the communication range for each node can notably improve the estimation performance but also significantly increase the communication overhead.

**Figure 11.**
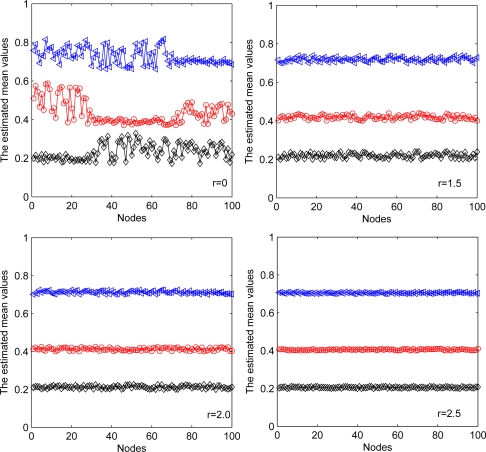
Estimated mean values versus radio range.

Different communication ranges for each node are also considered as well as the different exchange steps at each iteration for each node. In Figure 11, we show the performance of first estimated mean value for each sensor node with different communication ranges. Black line with diamond denotes the estimated mean value of the first component. Red line with circle denotes the estimated mean value of the second component. Blue line with triangle denotes the estimated mean value of the third component. The second estimated mean value has a similar result and is ignored here for simplicity. Without communication between sensor nodes, each estimated mean value has a relative accurate section, which represents reliable estimates of those nodes whose most observations come from the corresponding Gaussian component. Other nodes cannot properly estimate the parameters due to limited observations used. With diffusion between sensor nodes, the estimated mean values in all nodes approximate to their true values (the true values are 0.2, 0.4, and 0.7). With the increasing of the communication ranges, the estimation performance performance has been improved.

**Figure 12.**
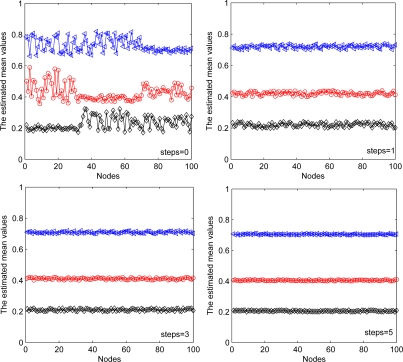
Estimated mean values versus steps of information exchange.

In addition, we consider the fixed communication range of each node with different steps of information exchange in the D-step of each iteration. From Figure 12, we can see that the estimation performance has been improved with increased exchange steps. These two simulations also show the trade-off between the estimation performance and communication overhead of sensor nodes.

## 6. Conclusions

This paper has presented a diffusion scheme of EM algorithm for distributed estimation of Gaussian mixtures in WSNs. In the E-step of this method, sensor nodes compute the local statistics by using local observation data and parameters estimated at the last iteration. A diffusion step is implemented over the communication networks after the E-step. In this step, the communication network is modeled as a connected graph, and each node exchanges local information only with its neighbors. In the M-step, the sensor nodes compute the estimation of parameter using the updated local statistics by the D-step at this iteration. Compared with the existing distributed EM algorithms, our proposed method can extensively reduce communication overhead for each sensor node while maintaining the estimation performance. In addition, we have shown that the proposed DEM algorithm can be considered as a stochastic approximation method to find the maximum likelihood estimation for Gaussian mixtures. Simulation results show good performance of our method.
